# Evaluating Guideline Alignment by Analyzing Patient Profiles of Elderly People with Type 2 Diabetes and Chronic Kidney Disease Treated or Not with SGLT2 Inhibitors

**DOI:** 10.3390/ph18060807

**Published:** 2025-05-27

**Authors:** Kyriaki Vafeidou, Ourania Psoma, Georgios Dimakopoulos, Evangelos Apostolidis, Anastasia Sarvani, Eleni Gavriilaki, Michael Doumas, Vassilios Tsimihodimos, Kalliopi Kotsa, Theocharis Koufakis

**Affiliations:** 1 Second Propaedeutic Department of Internal Medicine, Hippokration General Hospital, Aristotle University of Thessaloniki, 54642 Thessaloniki, Greece; kiriakivfd@gmail.com (K.V.); natasasar91@gmail.com (A.S.); elenicelli@yahoo.gr (E.G.); doumasm@auth.gr (M.D.); 2 Department of Internal Medicine, School of Medicine, University of Ioannina, 45110 Ioannina, Greece; raniapsoma@gmail.com (O.P.); vtsimi@uoi.gr (V.T.); 3 BIOSTATS, Epirus Science and Technology Park Campus, University of Ioannina, 45110 Ioannina, Greece; info@biostats.gr; 4 Department of Internal Medicine, General Hospital of Kozani, 50131 Kozani, Greece; vaggapo@gmail.com; 5 Division of Endocrinology and Metabolism and Diabetes Center, First Department of Internal Medicine, Medical School, Aristotle University of Thessaloniki, AHEPA University Hospital, 54636 Thessaloniki, Greece; kkalli@auth.gr

**Keywords:** SGLT2 inhibitors, type 2 diabetes, chronic kidney disease, elderly

## Abstract

**Background/Objectives:** Current guidelines for the management of type 2 diabetes (T2D) strongly recommend the use of sodium–glucose cotransporter 2 inhibitors (SGLT2is) in patients with chronic kidney disease (CKD) to alleviate cardiorenal risk. However, the implementation of this guidance in daily practice remains limited. In a real-world setting, we evaluated the frequency of SGLT2i use in elderly people with T2D and CKD and compared patient profiles between SGLT2i users and non-users. **Methods:** We retrospectively analyzed the medical records of individuals over 65 years of age followed in outpatient internal medicine clinics in Greece. Demographic and laboratory parameters, comorbidity profiles, and medication use were recorded and compared between the SGLT2i and non-SGLT2i groups. **Results:** The analysis included 135 patients with T2D and CKD, of whom the majority (57.8%) did not receive SGLT2i treatment. The patients in the SGLT2i group were younger (*p* = 0.006), had higher creatinine (*p* = 0.001) and hemoglobin (*p* = 0.001) values, and lower levels of uric acid (*p* = 0.025) than the participants not treated with SGLT2is. Heart failure rates were similar between the groups (*p* = 0.252). There was no difference in the use of renin–angiotensin–aldosterone system inhibitors (*p* = 0.210); in contrast, treatment with glucagon-like peptide 1 receptor agonists was more frequent in the group receiving SGLT2is compared to the group not treated with gliflozins (*p* = 0.002). **Conclusions:** Real-world data confirm the benefits of SGLT2i treatment for elderly people with T2D and CKD. However, our findings indicate that the use of gliflozins in this population of patients remains suboptimal, highlighting the need for greater vigilance among prescribers to align with existing guidelines.

## 1. Introduction

Type 2 diabetes (T2D) is currently one of the most common metabolic disorders worldwide and a major driver of life-threatening complications [1]. Among them, diabetes-related chronic kidney disease (CKD) is the leading cause of end-stage renal disease and is also associated with high cardiovascular (CV) risk, increased mortality rates, and poor quality of life [2]. The prevalence of CKD increases with age as elderly patients typically have multiple comorbidities, such as T2D, hypertension, and CV disease, that synergistically impair kidney function, while aging itself has a negative impact on renal homeostasis [3]. A recent study among patients with T2D and a mean age of 68 years followed in hospital-based diabetes clinics in Greece found that the prevalence of CKD in this population is alarmingly high, reaching 45% [4].

Sodium–glucose cotransporter 2 inhibitors (SGLT2is) are a relatively new class of drugs that have revolutionized the management of T2D. In addition to lowering blood glucose with a minimal risk of hypoglycemia and exerting positive effects on body weight and composition, SGLT2is have demonstrated impressive cardiorenal protective properties. Particularly in patients with T2D and CKD, gliflozins have been shown to reduce the risk of CV events, heart failure (HF), and renal outcomes, also attenuating the progressive decline in renal function seen in people with diabetes [5]. As a result, recent guidelines for the treatment of T2D strongly recommend the use of SGLT2is in patients with CKD, regardless of the quality of glycemic control and background therapy, to alleviate cardiorenal risk [6].

Despite the growing use of SGLT2is in daily practice, there is evidence that these agents are still under-prescribed, especially at the primary care level, even in high-risk patients who are expected to derive the greatest benefits from their use [7]. Fear of adverse events, underappreciation of the cardiorenal benefits of the class, and preference for a specialist to initiate therapy have been identified as potential explanations for the reluctance of general practitioners to prescribe SGLT2is [8]. There is evidence that older patients are more likely than younger ones to experience clinical inertia in the management of chronic diseases, including T2D [9]. This phenomenon could be attributed to safety concerns on the part of physicians, as elderly people are more vulnerable to adverse drug reactions, and there is also a lack of sufficient efficacy data given that this patient population is under-represented in randomized trials of new antidiabetic agents [10].

To date, there is scarce evidence on the implementation of guidelines on the management of elderly individuals with T2D and CKD. This study aimed to evaluate the frequency of SGLT2i use in this group of patients and to identify differences in patient profiles between SGLT2i users and non-users.

## 2. Results

The analysis included 135 patients with T2D and CKD, of whom the majority (*n* = 78, 57.8%) did not receive SGLT2i treatment. Among the participants treated with SGLT2is, 33 (57.9%) received dapagliflozin and 24 (42.1%) empagliflozin. Table 1 presents the demographic characteristics and comorbidities of the study participants.

**Figure 1:**
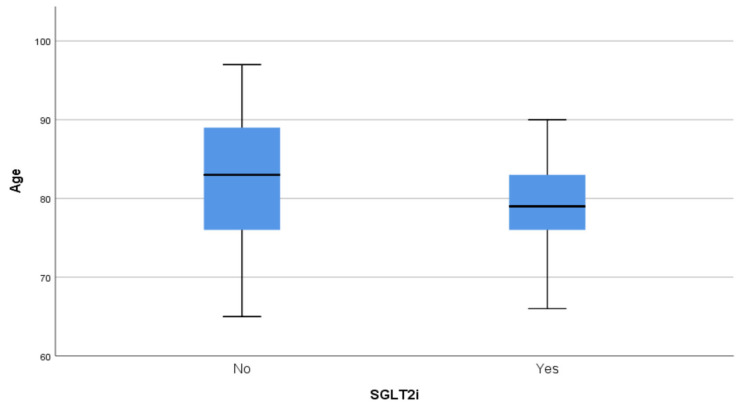
Distribution of age by treatment group. SGLT2i: sodium–glucose cotransporter 2 inhibitor.

In terms of demographic characteristics, there was no difference in the sex distribution between the groups (*p* = 0.164). On the contrary, patients who received SGLT2is were younger than those not treated with gliflozins (*p* = 0.006) (Figure 1).

Regarding comorbidities, there were no differences in the rates of HF (either with reduced or preserved ejection fraction) (*p* = 0.252), malignancy (*p* = 1.000), dementia (*p* = 0.096), obesity (*p* = 0.138), and metabolic-associated steatotic liver disease (*p* = 0.176) between the group treated with SGLT2is and the group without treatment. The FRAIL scale scores did not differ significantly between the groups (*p* = 0.341). Dyslipidemia was more frequent in patients receiving SGLT2is (*p* = 0.001), while liver cirrhosis was more frequent in patients who did not receive SGLT2is (*p* = 0.039), although the absolute number of cirrhosis cases in the cohort was small (*n* = 6).

**Figure 2:**
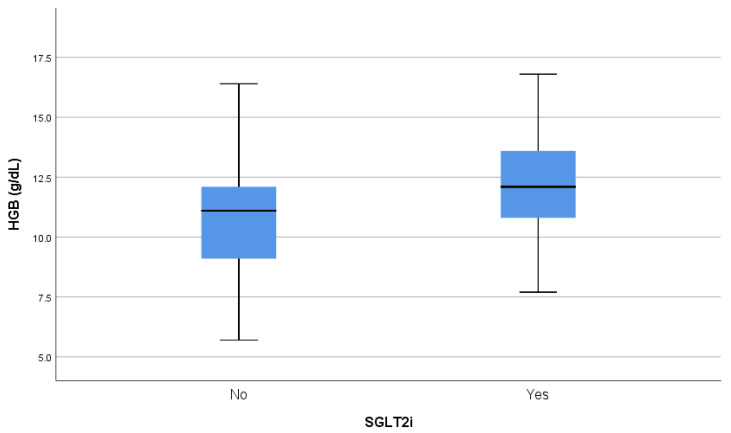
Hemoglobin values in SGLT2i users and non-users. HGB: hemoglobin; SGLT2i: sodium–glucose cotransporter 2 inhibitor.

In terms of markers of kidney function, we observed higher creatinine (*p* = 0.001) and urea (*p* = 0.038) levels and lower eGFR values (*p* = 0.059) among the SGLT2i-treated participants compared to those not receiving SGLT2is. The UACR levels were similar between the groups (*p* = 0.769). Despite worse renal function, the group treated with SGLT2is had higher hemoglobin (*p* = 0.001) (Figure 2) and hematocrit (*p* < 0.001) values compared to the group not receiving SGLT2is. The mean HbA1c levels were lower in the group treated with gliflozins (*p* = 0.010).

The potassium and calcium levels were higher (*p* = 0.005 and *p* = 0.036, respectively) and uric acid levels were lower (*p* = 0.025) in the group treated with gliflozins compared to patients who did not receive SGLT2i therapy. We did not find significant differences in the lipid profile or inflammation marker values (CRP: *p* = 0.754; ESR: *p* = 0.821) between the groups.

**Figure 3:**
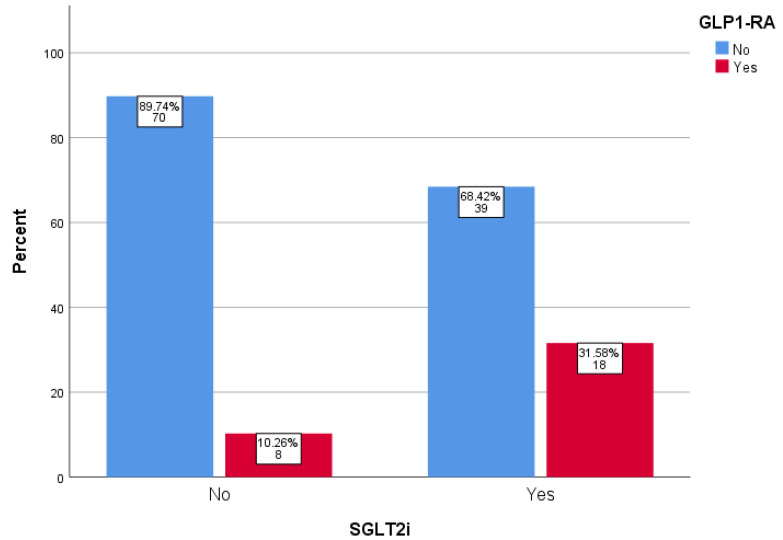
Distribution of GLP-1 RA therapy using SGLT2is. SGLT2i: sodium–glucose cotransporter 2 inhibitor; GLP-1 RA: glucagon-like peptide 1 receptor agonist.

In terms of medication use, there were no differences between the groups regarding RAASi (*p* = 0.210), insulin (*p* = 0.468), metformin (*p* = 0.235), sulphonylureas (*p* = 0.939), meglitinides (*p* = 1.000), dipeptidyl peptidase-4 inhibitors (*p* = 0.090), or thiazolidinediones (*p* = 0.236). In contrast, the use of glucagon-like peptide 1 receptor agonists (GLP-1 RAs) was more frequent in the group receiving SGLT2is compared to the group not treated with gliflozins (*p* = 0.002) (Figure 3). The polypharmacy rates were comparable between the groups (*p* = 0.451).

Table 2 presents the laboratory parameters and medications used by participants in the two study groups.

In the group of patients receiving SGLT2is, there was no statistical difference in any of the investigated parameters (demographic data, medications, or comorbidities) between those receiving empagliflozin and those receiving dapagliflozin.

## 3. Discussion

To the best of our knowledge, this study is the first attempt to capture the real-world picture of SGLT2i use in elderly patients with T2D and CKD in Greece. We observed distinct patient profiles between SGLT2i users and non-users, as well as a significant discordance between everyday clinical practice and current guidelines for the treatment of CKD in the context of T2D.

Following the publication of large-scale randomized trials that proved the CV- and renal-protective properties of SGLT2is, their prescription rate increased but remained suboptimal. In contrast, the prescription rates for dipeptidyl peptidase-4 inhibitors remained stable, despite the lack of evidence supporting the cardiorenal benefits of this class of drugs [11]. Additionally, significant disparities in prescription rates were observed between different patient populations. Specifically, elderly patients and patients with lower incomes were reported to receive SGLT2is less frequently [12]. Patients with a known history of HF or diabetes-related complications were less likely to receive SGLT2is, despite the recommendations supporting their use. The same prescription pattern is reported in patients with a history of recurrent urinary tract infections [11,12]. On the contrary, younger age, higher levels of HbA1c, lower UACR, and the use of eGFR and RAASis were associated with higher rates of SGLT2i prescription [13]. In 2017, only 5% of eligible patients received SGLT2 inhibitors according to ADA recommendations [11,12,14].

Our findings are in line with a recently published report by Hösli et al. that analyzed data from outpatients enrolled in the Swiss Diabetes Registry [15]. In contrast to our work, their study did not focus on elderly patients, resulting in a mean age of participants that was approximately 10 years lower than our study population. The authors found that two out of three patients with T2D and CKD in Switzerland did not receive SGLT2i treatment, and for the majority (66%), there was no obvious reason for this deviation from the guidelines. Together with the findings of the present study, these data highlight that despite convincing evidence of the benefits of these new antidiabetic agents, clinical inertia is highly prevalent in daily practice, regardless of country-specific differences in prescription and reimbursement policies.

Differences in patient profiles between participants treated or not with SGLT2is provide some indirect evidence on factors associated with the under-prescription of these agents in people with T2D and CKD. Younger participants receiving SGLT2is had more advanced CKD, as indicated by lower eGFR values compared to the group not receiving gliflozin therapy. The inception of SGLT2i treatment has been associated with an acute, reversible decrease in eGFR values, typically observed during the first weeks of treatment, with subsequent partial recovery of creatinine levels at week 12, ultimately followed by an attenuation of the eGFR decreasing compared to the control group after 52 weeks [16]. However, this should not be the case with the participants in the present study since, according to the inclusion criteria, they had been receiving SGLT2i treatment for more than 12 months. Furthermore, considering the similar UACR values in the two groups and the positive effect that SGLT2is have on albuminuria, it is very possible that the group of patients who received SGLT-2is had significantly higher pretreatment values of UACR than the comparison group. Therefore, our observations imply that prescribers prefer to withhold SGLT2i treatment until CKD has progressed significantly. Although SGLT2is have been shown to retain renal-protective properties even in stage 5 CKD patients with T2D [17], an approach of “the earlier the better” has been proposed with respect to their introduction to the therapeutic regimen [18]. In support of this perspective, data from the DECLARE-TIMI 58 trial suggest that dapagliflozin mitigated kidney function decline even in patients with low baseline renal risk, indicating a role for SGLT2is in the primary prevention of adverse kidney outcomes [19].

The higher mean age of patients in the group not receiving SGLT2is confirms recently published findings from Singapore on the hesitation of primary care physicians to prescribe these agents in those belonging to the “oldest old” category [20]. Euglycemic diabetic ketoacidosis (DKA), genital infections, the deterioration of sarcopenia, and volume depletion, particularly in the context of co-administration with loop diuretics, have been the main safety concerns regarding the use of SGLT2is in older people with CKD [10]. A meta-analysis of 39 randomized trials found that the older the age, the higher the risk of SGLT2i-induced DKA [21]. On the other hand, a recent study suggests that despite experiencing smaller reductions in HbA1c values compared to younger patients, older people derive greater benefits in terms of heart and kidney protection from SGLT2i therapy [22]. In addition, Lunati et al. [23] showed that starting SGLT2i treatment in patients with T2D after the age of 70 is well tolerated and safe, although some caution, particularly with respect to urinary tract infections and the worsening of renal function, is needed in more fragile subjects.

Among participants not receiving treatment with SGLT2is, we also observed a lower rate of GLP-1 RA use, which probably explains the higher HbA1c values in this group. GLP-1 RA have demonstrated significant nephroprotective properties in patients with T2D [24]. However, data show that in T2D, there is a considerable delay in the initiation of injectable therapies despite poor glycemic control, which leads to an increased risk of complications [25]. In contrast to the above, we found no differences between the groups in the use of RAASis, which, however, was suboptimal. As the nephroprotective effects of RAASis have been well established for decades [26], it may take longer for the recently discovered renoprotective properties of the newer classes of antidiabetic drugs to fully integrate into clinical practice.

In a real-world setting, our findings confirm the important benefits of gliflozins for people with CKD. In particular, we observed higher levels of hemoglobin and hematocrit in the group treated with SGLT2is compared to the group without gliflozin therapy, despite worse renal function. There is robust evidence that anemia and delay in its treatment are associated with an increased risk of CV events, HF, and all-cause death among patients with CKD [27]. Treatment with SGLT2is has been shown to ameliorate anemia through various mechanisms, including increased erythropoietin production and erythropoiesis, as well as the improved regulation of iron homeostasis [28]. In fact, these actions, which enhance tissue oxygenation and metabolism, have been suggested to be the strongest mediators of the renoprotective properties of SGLT2is observed in large cardiovascular outcome trials [29]. Furthermore, we found significantly lower levels of serum uric acid in the group of participants treated with SGLT2is. Higher levels of uric acid have been associated with a faster decline in renal function and a higher risk of kidney failure [30]. SGLT2is are believed to reduce serum uric acid by normalizing nutrient signaling, decreasing purine synthesis, and promoting renal urate excretion [31]. La Grotta et al. [32] showed that the potential of SGLT2is to alleviate low-grade inflammation, a key player in the development of renal dysfunction, is mediated by their uric acid-lowering effects.

The findings of the present study should be interpreted in light of specific strengths and limitations. Taking into account regional variation in health care services, it is important to obtain country-specific data on the implementation of guidelines in clinical practice [33]; therefore, our study provides real-world evidence on the suboptimal use of SGLT2is in Greek patients with T2D and CKD and patient-specific factors that could be related to the decision of physicians to prescribe these drugs or not. The exclusion of patients who had contraindications or adverse events related to SGLT2is re-emphasizes that clinical inertia was one of the main reasons for non-treatment. On the other hand, the retrospective, cross-sectional nature of the study is an important source of selection and other biases and is therefore unable to establish causal associations. Although guidelines are evidence-based statements that provide clinicians with helpful guidance for daily practice, they cannot substitute the clinical judgment necessary to assess interpatient variability—a factor that could not obviously be incorporated into our analysis. Detailed anthropometric data, such as waist circumference or muscle volume, that were not available for our cohort would help complete the profiling of patients with T2D and CKD treated with SGLT2is or not. However, we compared obesity rates between groups and found no differences. Finally, factors related to access to health care, such as the socioeconomic status of participants, were not evaluated, since there is wide access to antidiabetic treatments at little or no cost in the Greek health care system, even for those without social security.

In conclusion, real-world data from Greece confirm the benefits of SGLT2i treatment for elderly people with T2D and CKD. However, our findings indicate that the use of gliflozins in this population of patients remains suboptimal, highlighting the need for greater vigilance among prescribers to align with existing guidelines. We replicate previous research from other parts of the world showing the presence of clinical inertia that compromises patient outcomes in the field of cardiometabolic medicine. Given the relatively high mean age of our study sample, future prospective studies should investigate whether clinical inertia with respect to the use of SGLT2is in this population negatively affects quality of life and increases the risk of complications, particularly for “hard” endpoints such as renal death and time to initiate dialysis.

## 4. Materials and Methods

### 4.1. Study Design

For this retrospective observational study, we analyzed the medical records of patients followed in two public outpatient internal medicine clinics in northern and western Greece, respectively. Data collection took place between November 2024 and February 2025. The study inclusion criteria were as follows: a T2D diagnosis from a physician according to the guidelines of the American Diabetes Association (ADA) [34]; age ≥ 65 years; presence of CKD defined as an estimated glomerular filtration rate (eGFR) <60 mL/min/1.73 m^2^ and/or urine albumin–creatinine ratio (UACR) > 30 mg/g persistently for at least 3 months; and consecutive SGLT2i treatment for at least 12 months (only for the group prescribed SGLT2i). We primarily took into account the KDIGO guidelines [35] based on previously published studies with a similar design and objectives [15]. However, as can be seen in Table 3, the recommendation of prescribing SGLT2is as first-line treatment for diabetic kidney disease is uniform across guidelines of different scientific societies [13,35,36,37,38].

**Figure 4:**
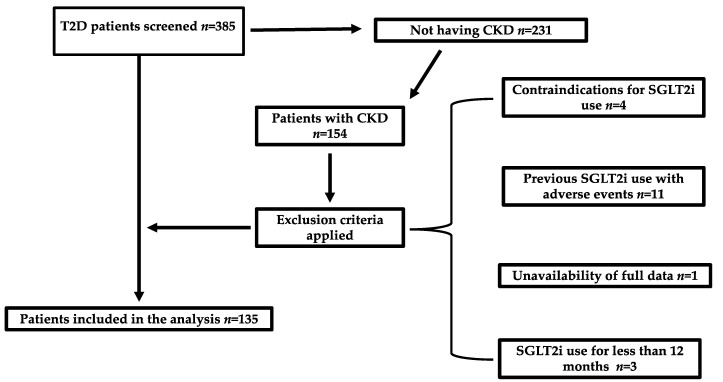
Flowchart of the study. T2D: type 2 diabetes; CKD: chronic kidney disease; SGLT2i: sodium–glucose cotransporter 2 inhibitor.

The exclusion criteria were a history of diabetes other than type 2; the existence of contraindications to the use of SGLT2is, such as eGFR values < 20 mL/min/1.73 m^2^ or severe disorders of the urinary system; previous treatment with SGLT2is that was interrupted due to adverse effects or intolerance; and unavailability of the necessary clinical or laboratory data at the time of inclusion in the study. Figure 4 presents the study selection flowchart.

For patients who met the above criteria, demographic and laboratory parameters were recorded, as well as a complete history of comorbidities and medication use. More specifically, the parameters of interest included sex, age, type of SGLT2i used, concomitant treatment with hypoglycemic agents and renin–angiotensin–aldosterone system inhibitors (RAASis), complete blood count, fasting glucose, glycated hemoglobin (HbA1c), serum values of urea and creatinine, c-reactive protein (CRP), erythrocyte sedimentation rate (ESR), UACR, and lipid and electrolyte profiles. The eGFR was calculated using the 2021 CKD-EPI equation [35]. Anthropometric parameters included body weight and height. Height was measured with a Holtain wall stadiometer. Body weight was recorded to the nearest 0.1 kg using a calibrated computerized digital balance (K-Tron P1-SR, Onrion LLC, Bergenfield, NJ, USA). Each participant was barefoot and dressed lightly during the assessment. The BMI was calculated by dividing the weight of a participant in kilograms by their height in meters squared. Blood samples were drawn in the morning, after a 12 h overnight fast, via antecubital venipuncture. Biochemical parameters were determined using the COBAS 8000 automated analyzer system (D-68298; Roche Diagnostics, Mannheim, Germany). Polymedication was defined as the daily use of ≥4 drugs [39], and the frailty status of the participants was evaluated with the FRAIL questionnaire, which is a reliable and easy-to-use tool to detect frailty in older individuals and has been validated in the Greek language and population [40]. FRAIL scale scores range from 0 to 5 (0 = robust health status; 1–2 = pre-frail; 3–5 = frail).

### 4.2. Statistical Analysis and Ethical Considerations

All outcome measures were described using means and standard deviations, medians, or percentages. The normality of the variables examined was assessed using the Kolmogorov–Smirnov criterion to specify the tests that would lead to reliable inference regarding the differences between patients receiving and not receiving SGLT2is. In most cases, the normality hypothesis was rejected, while in a few cases, there was borderline non-significance. A non-parametric approach was therefore adopted to examine the differences in interest. The differences between the two groups were evaluated using the Mann–Whitney U test or the chi-squared test when the use of SGLT2is was correlated with gender, comorbidities, or medications. Analysis was carried out using SPSS v. 26.0, and the statistical significance was set to 0.05 in all cases. Differences in the distribution of age and hemoglobin according to the use of SGLT2is were represented using comparative boxplots.

The study was carried out according to the principles of the Declaration of Helsinki and its subsequent amendments. All study participants provided their informed written consent prior to enrolment in the study, and the study protocol was approved by the institutional review board of the Aristotle University of Thessaloniki (approval number 1/12 November 2024).

## Figures and Tables

**Table 1:** Comparison of demographic features and comorbidity rates between the SGLT2i group and the non-SGLT2i group.

Variable	SGLT2i Group	Non-SGLT2i Group	*p*-Value
Demographics			
Age (years)	78.65 ± 5.94 (79)	82.03 ± 7.53 (83)	**0.006**
Male gender (%)	57.9	44.9	0.164
Comorbidities			
Heart failure (%)	60.0	76.0	0.252
Hypertension (%)	80.7	75.6	0.535
Dyslipidemia (%)	75.4	46.2	**0.001**
Hyperuricemia (%)	22.8	26.9	0.586
Obesity (%)	27.8	25.1	0.138
Atrial fibrillation (%)	21.0	26.9	0.433
Ischemic heart disease (%)	29.8	17.9	0.105
Stroke/TIA (%)	17.5	14.1	0.586
COPD/lung disease (%)	12.3	15.4	0.608
MASLD (%)	53.5	57.8	0.176
Liver cirrhosis (%)	0.0	7.7	**0.039**
Malignancy (%)	19.3	19.2	1.000
Dementia (%)	5.3	14.1	0.096
Autoimmune disease/arthritis (%)	5.3	3.8	0.697
Mental health disorder (%)	7.0	2.6	0.240
FRAIL scale	3.7 ± 1.2	3.3 ± 0.6	0.341

SGLT2i: sodium–glucose co-transporter-2 inhibitor; COPD: chronic obstructive pulmonary disease; MASLD: metabolic dysfunction-associated steatotic liver disease; TIA: transient ischemic attack. Bold *p*-values denote statistical significance. Values are presented as percentages or means ± standard deviation (medians).

**Table 2:** Comparison of pharmacological treatments and laboratory parameters between the SGLT2i group and the non-SGLT2i group.

Variable	SGLT2i Group	Non-SGLT2i Group	*p*-Value
Pharmacological treatment			
Metformin (%)	47.4	57.7	0.235
Insulin (%)	38.6	32.1	0.468
GLP-1 RA (%)	31.6	10.3	**0.002**
DPP4i (%)	40.4	55.1	0.090
Sulphonylureas (%)	21.1	20.5	0.939
Thiazolidinediones (%)	12.3	6.4	0.236
Meglitinides (%)	0.0	1.3	1.000
RAASi (%)	75.4	65.4	0.210
Polypharmacy ≥ 4 drugs (%)	69.2	73.4	0.451
Laboratory parameters			
HbA1c (%)	7.69 ± 1.56 (7.25)	8.29 ± 10.61 (6.9)	**0.010**
Fasting glucose (mg/dL)	150.51 ± 62.33 (131)	135.20 ± 52.33 (123)	0.156
Urea (mg/dL)	86.53 ± 45.18 (76.8)	71.25 ± 30.13 (65)	0.038
Creatinine (mg/dL)	1.74 ± 0.44 (1.74)	1.52 ± 0.39 (1.46)	**0.001**
eGFR (mL/min/1.73 m^2^)	37.02 ± 10.14 (34)	40.23 ± 10.01 (38.5)	0.059
UACR (mg/g)	101.00 ± 133.56 (31)	103.50 ± 139.30 (103.5)	0.769
Total cholesterol (mg/dL)	135.33 ± 36.64 (135)	138.20 ± 54.21 (131)	0.849
Triglycerides (mg/dL)	160.45 ± 103.20 (128.5)	132.77 ± 84.37 (110)	0.164
LDL-C (mg/dL)	65.39 ± 29.79 (69)	73.49 ± 45.83 (67)	0.530
HDL-C (mg/dL)	40.28 ± 12.13 (41.65)	38.93 ± 13.64 (38.8)	0.306
Uric acid (mg/dL)	6.33 ± 1.85 (6.3)	7.43 ± 2.50 (6.91)	**0.025**
Hemoglobin (g/dL)	12.32 ± 2.01 (12.1)	11.14 ± 3.43 (11.1)	**0.001**
Hematocrit (%)	38.06 ± 5.76 (37.7)	33.42 ± 7.29 (33.6)	**<0.001**
Sodium (mmol/L)	138.06 ± 5.26 (138)	138.99 ± 4.71 (139)	0.160
Potassium (mmol/L)	4.54 ± 0.69 (4.5)	4.17 ± 0.67 (4.09)	**0.005**
Calcium (mg/dL)	9.43 ± 0.60 (9.4)	9.15 ± 0.68 (9.1)	**0.036**
Phosphorus (mg/dL)	3.93 ± 1.23 (3.64)	3.78 ± 0.94 (3.61)	0.553
SGOT (IU/L)	31.24 ± 54.37 (20.8)	37.01 ± 80.94 (21.1)	0.637
SGPT (IU/L)	24.09 ± 22.90 (17.65)	22.81 ± 47.24 (12.5)	**0.027**
CPK (IU/L)	80.33 ± 86.65 (45.6)	148.76 ± 330.56 (63.1)	0.216
CRP (mg/dL)	8.17 ± 10.26 (4.76)	6.09 ± 10.26 (3.27)	0.754
Ferritin (ng/mL)	182.26 ± 210.77 (113)	246.68 ± 530.41 (117)	0.607
ESR (mm/h)	61.48 ± 35.46 (61)	60.36 ± 35.17 (50)	0.821

SGLT2i: sodium–glucose co-transporter-2 inhibitor; GLP-1 RA: glucagon-like peptide-1 receptor agonist; DPP4i: dipeptidyl peptidase-4 inhibitor; RAASi: renin–angiotensin–aldosterone system inhibitor; HbA1c: glycated hemoglobin; eGFR: estimated glomerular filtration rate; UACR: urinary albumin-to-creatinine ratio; LDL/HDL: low-/high-density lipoprotein cholesterol; SGOT/SGPT: liver transaminases; CPK: creatine phosphokinase; CRP: C-reactive protein; ESR: erythrocyte sedimentation rate. Bold *p*-values denote statistical significance. Values are presented as percentages or means ± standard deviation (medians).

**Table 3:** Comparison of recommendations of various scientific societies on SGLT2i use in diabetic kidney disease.

Guidelines	Recommendations on SGLT2is	eGFR Threshold	Elderly-Specific Considerations
KDIGO 2024	SGLT2i use for all patients with T2D and CKD with eGFR ≥20 mL/min/1.73 m^2^	Initiate if eGFR ≥ 20 mL/min/1.73 m^2^ and continue use until initiation of dialysis or transplantation	No specific recommendations for elderly patients; individualized treatment is emphasized
KDOQI 2025	SGLT2is as first-line therapy for renal and cardiovascular protection in patients with eGFR ≥ 20 mL/min/1.73 m^2^ and UACR ≥200 mg/g	Initiate if eGFR ≥ 20 mL/min/1.73 m^2^ and continue use until initiation of dialysis or transplantation	No specific recommendations for elderly patients; individualized treatment is emphasized
ADA 2024	SGLT2i use to reduce CKD progression and cardiovascular events in patients with T2D and CKD with eGFR ≥ 20 mL/min/1.73 m^2^, regardless of albuminuria level	Initiate if eGFR ≥ 20 mL/min/1.73 m^2^ and continue use until initiation of dialysis or transplantation	No specific recommendations for elderly patients; individualized treatment is emphasized
EASD 2022	SGLT2i use in patients with CKD and eGFR ≥ 20 mL/min/1.73 m^2^ and UACR > 30 mg/g to reduce MACEs and HF and improve kidney outcomes	Initiate if eGFR ≥ 20 mL/min/1.73 m^2^ and continue use until initiation of dialysis or transplantation	No specific recommendations for elderly patients; individualized treatment is emphasized
ERA 2023	SGLT2i use for cardiorenal protection in patients with T2D and CKD with eGFR ≥ 20–60 mL/min/1.73 m^2^ or UACR > 30 mg/g	Initiate if eGFR ≥ 20 mL/min/1.73 m^2^ and continue use until initiation of dialysis or transplantation	No specific recommendations for elderly patients; individualized treatment is emphasized

SGLT2i: sodium–glucose cotransporter 2 inhibitor; T2D: type 2 diabetes; CKD: chronic kidney disease; eGFR: estimated glomerular filtration rate; MACEs: major adverse cardiovascular events; UACR: urine albumin–creatinine ratio.

## Data Availability

The data presented in the study are available from the corresponding author upon request. The data are not publicly available due to privacy restrictions of the Greek National Health System.
